# Are anxiety disorders a prelude to delusional disorder? A systematic review

**DOI:** 10.1192/j.eurpsy.2021.506

**Published:** 2021-08-13

**Authors:** A. Alvarez Pedrero, A. González-Rodríguez, A. Guàrdia, L. Delgado, G.F. Fucho, S. Acebillo, J.A. Monreal, J. Labad, D. Palao Vidal

**Affiliations:** 1 Mental Health, Parc Taulí University Hospital. Autonomous University of Barcelona (UAB). I3PT, Sabadell, Spain; 2 Mental Health, Parc Taulí-University Hospital, Sabadell, Spain; 3 Mental Health, Parc Tauli University Hospital, Sabadell, Spain; 4 Mental Health, Hospital of Mataró. Consorci Sanitari del Maresme. CIBERSAM., Mataró, Spain

**Keywords:** Delusional disorder, Anxiety, panic attack, Treatment

## Abstract

**Introduction:**

Prevalence rates of panic attacks have been reported to be around 24-63% in psychotic patients. Common underlying biological substrates for panic and paranoia have been proposed, suggesting that delusional disorder (DD) may be preceded by the development of anxiety disorders.

**Objectives:**

The main objective of this study was to investigate anxiety comorbidity in DD. As a second objective, we set ourselves to know prescription rates for the use of antidepressants and benzodiazepines in anxiety disorders in the context of DD.

**Methods:**

A systematic literature search was performed using PubMed (1980- September 2020) according to the PRISMA guidelines. The following search terms were used: (delusional disorder) AND (anxiety OR anxiety disorder OR anxi*). Research studies and case reports were included if they met the following criteria: DD diagnosis (DSM, ICD), publication in peer-review journal and investigations containing information on anxiety comorbidity in DD.

**Results:**

Four studies fulfilled our criteria, including 155 patients: 65 (42%) women, mean age 42.7 years (SD:14.96). Thirty-three of the 155 patients (21.29%) presented at least one comorbid anxiety disorder: 14 specific phobias, 9 panic attacks, 5 social phobias and 2 agoraphobias. Treatment was not reported for many patients (n= 28). Four patients received fluoxetine and 1 patient benzodiazepines. All of them showed partial improvement of symptoms.

**Conclusions:**

Less than a third of DD patients showed an anxiety disorder. The effectiveness of antidepressant and benzodiazepine treatment has been poorly described. Future studies may be focused on the investigation of preceding comorbid anxiety disorders in patients with DD.

